# Would a thermal sensor improve arm motion classification accuracy of a single wrist-mounted inertial device?

**DOI:** 10.1186/s12938-019-0677-7

**Published:** 2019-05-07

**Authors:** Jordan Lui, Carlo Menon

**Affiliations:** Menrva Research Group, Schools of Mechatronic System and Engineering Science, Simon Fraser University, Metro Vancouver, BC Canada

**Keywords:** Rehabilitation, Telerehabilitation, Home rehabilitation, Wearable, Motion classification, Infrared motion tracking, Inertial motion tracking

## Abstract

**Background:**

Inertial Measurement Unit (IMU)-based wearable sensors have found common use to track arm activity in daily life. However, classifying a high number of arm motions with single IMU-based systems still remains a challenging task. This paper explores the possibility to increase the classification accuracy of these systems by incorporating a thermal sensor. Increasing the number of arm motions that can be classified is relevant to increasing applicability of single-device wearable systems for a variety of applications, including activity monitoring for athletes, gesture control for video games, and motion classification for physical rehabilitation patients. This study explores whether a thermal sensor can increase the classification accuracy of a single-device motion classification system when evaluated with healthy participants. The motions performed are reproductions of exercises described in established rehabilitation protocols.

**Methods:**

A single wrist-mounted device was built with an inertial sensor and a thermal sensor. This device was worn on the wrist, was battery powered, and transmitted data over Bluetooth to computer during recording. A LabVIEW Graphical User Interface (GUI) instructed the user to complete 24 different arm motions in a pre-randomized order. The received data were pre-processed, and secondary features were calculated on these data. These features were processed with Principal Component Analysis (PCA) for dimensionality reduction and then several machine learning models were applied to select the optimal model based on speed and accuracy. To test the effectiveness of the scheme, 11 healthy subjects participated in the trials.

**Results:**

Average personalized classification model accuracies of 93.55% were obtained for 11 healthy participants. Generalized model accuracies of 82.5% indicated that the device can classify arm motions on a user without prior training. The addition of a thermal sensor significantly increased classification accuracy of a single wrist-mounted inertial device, from 75 to 93.55%, (*F*(1,20) = 90.53, *p* = 7.25e−09).

**Conclusion:**

This study found that the addition of the thermal sensor improved the classification accuracy of 24 arm motions from 75 to 93.55% for a single-device system. Our results provide evidence that a single device can be used to classify a relatively large number of arm motions from arm rehabilitation protocols. While this study provides a conceptual proof-of-concept with a healthy population, additional investigation is required to evaluate the performance of this system for specific applications, such as activity classification for physically affected stroke survivors undergoing home-based rehabilitation.

**Electronic supplementary material:**

The online version of this article (10.1186/s12938-019-0677-7) contains supplementary material, which is available to authorized users.

## Introduction and background

Motion tracking and classification is a topic of interest with applications in medicine, military, security industry, sports, and development of human–machine interfaces [1, 2]. Wearable sensors have been applied in rehabilitation for detecting freezing in the gait cycle of Parkinson’s patients, detecting epileptic seizures, cognitive function assessment in older adults, and motion classification of stroke exercise [3]. Systems for motion classification usually track motion through use of body-worn sensors (wearables) or through camera vision systems placed in the room [4]. Vision systems have been proposed using conventional cameras, motion capture cameras, and depth cameras such as the Microsoft Kinect camera [5], but introduction of these technologies in the home can introduce concerns about unauthorized access to personal health information that is recorded or displayed in a private setting [6]. Camera solutions also require adequate space to see the participant in the room; these can be difficult to implement outside of a lab setting [4, 7]. Wearable sensor systems frequently use accelerometer or gyroscope sensors, or combined units called inertial measurement units (IMU). Systems with multiple separate devices are frequently proposed for sensing the orientation of each limb, allowing the position of each limb to be obtained by solving the relevant kinematics equations.

Bao et al. [8] report 84% classification accuracy on user annotated data of 20 motions with 5 devices placed on the arms, legs, and waist. These motions included walking, sitting, running, brushing teeth, eating, and biking. Zinnen et al. [9] report 93% classification accuracy for 20 motions with a 5 device system, and 86% accuracy when 2 wrist devices are used. These motions included opening and closing various doors of a car and a writing task. Zhang et al. [10] proposed a 2-device system that classified 4 arm motions with 97.2% accuracy. A multi-device system would generally be more complex to don than a single-device system, especially when units are placed on multiple limbs or a calibration between devices is required. A single-device system would be preferred where possible since it would be simpler to don and configure.

Several single-device systems have been proposed which track ambulation (running and walking) and poses (standing and sitting). For example, Zhang et al. [11] reported 96.1% accuracy for 9 activities that included walking, running, sitting, and standing with healthy participants. In another study, Khan et al. [12] reported 97.9% accuracy on 15 motions with a chest-based device for various activities such as lying, sitting, standing, and transitions between these activities. Ravi et al. [13] classified 8 ambulation motions with 99.8% accuracy using a single device situated at pelvis. These systems, however, would likely be unable to classify arm rehabilitation exercises since the sensors were placed on the torso or waist.

A few single-device systems have been proposed for classification of arm motions for healthy and stroke-survivor populations. Tseng et al. [14] reported a single-device system with 93.3% accuracy for classifying 3 motions related to opening a door in a study with 5 healthy participants. Panwar et al. [15] conducted studies of motion classification for 3 drinking motions with 4 healthy participants and reported 99.8% accuracy when using a convolutional neural network (CNN). Biswas et al. [16] reported 91–99% accuracy for 3 reaching arm motions with 4 healthy participants and 70–85% accuracy with stroke survivors. Yang et al. [17] achieved 95% classification accuracy of 8 activities using a neural network; 4 of those actions involved some arm motion, while 4 other motions were primarily ambulation activities. Zhang et al. [18] proposed a single IMU system for classification of 6 arm motions with 14 stroke participants and reported up to 99.4% and 98.8% accuracy with a novel model and a Support Vector Machine (SVM) model, respectively. These 6 motions represented 4 of 7 degrees of freedom of the human arm: shoulder extension/flexion, shoulder adduction/abduction, forearm pronation/supination, and elbow extension/flexion. Movements representing shoulder abduction, wrist flexion/extension, and wrist adduction/abduction were not represented. In the author’s literature review, the highest number of arm motions classified in a single-device system was 8 motions. The authors’ literature review found that IMU-based single-device systems were used to classify a maximum of 8 arm motions, arguably due to a difficulty in classifying higher numbers of arm motions with a single inertial sensor. However, detecting multiple arm motions might be of interest for different populations, such as patients undergoing physical rehabilitation. In the example of stroke survivors, rehabilitation exercise manuals, such as Chedoke-McMaster Stroke Assessment [19], Chedoke Arm and Hand Activity Inventory (CAHAI) [20], and Graded Repetitive Arm Supplementary Program (GRASP) [21], describe exercises for most human arm degrees of freedom and each protocol lists more than 8 motions, and therefore it is imperative to explore other solutions to realize classification of higher numbers of arm movements. In this study, we propose to explore the possibility of incorporating additional sensor data into a single-device platform as one solution to the current limitation.

Increasing the number of motions that can be tracked could improve tracking in home-based rehabilitation, which is an attractive alternative to clinical rehabilitation when patients lack transportation to access treatment [3, 22] or because patients are not comfortable performing exercise in a facility [22]. Additionally, home-based rehabilitation can be shown to reduce rehabilitation healthcare costs by 15% [23]. Home-based rehabilitation has strong promise for improving health outcomes but a key challenge exists: patients report difficulty adhering to the regimen and also have difficulty reporting accurately on their exercise [24, 25]. Telerehabilitation specifically could allow a clinician to assess a patient’s progress via a live call or through review of previously recorded exercise data and could lower the barriers to rehabilitation access for people in rural areas and people with severe physical impairment. Systems for telerehabilitation have been proposed which use camera teleconference systems, robotic rehabilitation devices, and home objects which have been instrumented with sensors [26–28]. In tracking unsupervised exercise, we must consider several metrics in order to describe the quantity and quality of motion performed. Accurate reporting of exercise consists of several aspects: intensity, quantity, frequency, duration, and quality of motion [29]. Quantity, frequency, and duration describe the dose of exercise received by the patient, while quality (or correctness) describes how similarly the patient completes the motion in relation to some specified standard. Quality of motion performed is important in rehabilitation since it influences the speed of patient recovery and improper form in exercise can delay healing progression and even increase risk of patient injury [30]. However, patients are sometimes unable to self-report accurately to their clinician on both the quantity and quality of exercise motions completed [30].

The authors have previously combined infrared (IR) sensors and inertial sensors into single-device wrist-mounted wearables with the goal of increasing the variety of sensor data available and thus possibly improving the device’s ability to classify arm motions. The motivation was that a thermal sensor might provide valuable information about the temperature value of objects in the sensor field of view, and thus might provide insight into the relative orientation between the user’s wrist and body. In a preliminary study, the authors first built a prototype system that used an IMU, passive thermal sensor, and IR distance sensor to classify 4 motion classes with 88% accuracy in Lui et al. [31]. A subsequent study into position classification using an inertial and thermal sensor system indicated that the passive thermal sensor might be a strong contributor to accuracy, while the IR distance sensor was not a significant contributor to accuracy [32]. Analysis of these results also indicated that orienting the thermal sensor 45 degrees from a normal vector at the wrist inwards towards the torso provided better performance than the thermal sensor oriented 45 degrees from the normal vector outwards away from the torso.

This study aimed to verify if the addition of a thermal sensor could increase classification accuracy of a single wrist-mounted device. This was completed by performing a study with 11 healthy participants, using the device to classify 24 motions. The motions were selected from established rehabilitation protocols to characterize performance in healthy participants in view of future studies that will evaluate the performance of this device with a stroke-survivor population.

## Materials and methods

### System design

#### Hardware

The system is a single unit device with a MPU9250 IMU and Omron D6T thermal sensor. The device is battery powered and continuously acquires sensor data when powered. When the device is powered by a 1200-mAh lithium polymer battery, the battery life is about 15 h. When a recording device like a laptop computer is connected via Bluetooth connection, sensor data are continuously transmitted. Sensor data interpretation and motion classification is performed on the computer. It is mounted on a soft foam band with straps, allowing it to be comfortably attached to the body.

#### Sensor data

The device has two main sensors: The MPU9250 IMU and Omron D6T passive thermal sensor. The MPU9250 [33] is an IMU with a 3-axis accelerometer, 3-axis gyroscope, and 3-axis magnetometer. The sensor data were transmitted to a recording computer at a 12-Hz frequency; similar studies have reported that signal data frequency of 10-Hz is sufficient to detect arm motions [34]. The Omron D6T [35] is a passive infrared thermal sensor that reports a 4-by-4 grid of temperature readings in its 45-degree by 45-degree field of view (FOV) and can greatly aid in detecting the presence of a human body, even through thick clothing. When mounted on the arm, the thermal sensor provides temperature data that can provide insight into the relative orientation between the user’s wrist and body.

### Experimental protocol

#### Participants

11 healthy participants (6 females, 5 males) participated in this study. Their median age was 28 ± 6 years. All were right handed. All signed a consent form for this study that was approved by Simon Fraser University Research Ethics Board.

#### Protocol

Each participant was seated at a table and performed 24 motions with their left hand; these motions are described in Table 1. The motions were derived or adapted from rehabilitation protocols which are currently used for unilateral upper extremity rehabilitation: Chedoke-McMaster Assessment [19], CAHAI, [20], GRASP, [36], and Bobath [18]. A LabVIEW virtual instrument (VI) program was created to conduct the study; the program received data from the device, saved it to the computer disk, and had a graphical user interface (GUI) which prompted the user to complete each action with an on-screen message. Motions were completed in 10 repetitions; the task order for each repetition was pre-generated with a pre-determined random shuffle. A test supervisor was present to monitor the testing and managed the data acquisition in accordance with university research ethics.

**Table 1:** 24 protocol motions

#	Motion	References	Exercise notes
1	Bobath handshake	[18]	Grasp hands together and raise arms upwards
2	Straight arm press	[18]	Place palm on chair and straighten arm
3	Horizontal shoulder extension	[18]	Start with hand on opposite shoulder and rotate shoulder outwards 180°
4	Elbow to nose	[18]	With hand on opposite shoulder, flex shoulder to bring elbow up to nose
5	Touch shoulder	[18]	Reach from table to place hand on opposite shoulder
6	Supinate	[18]	Arm on table, supinate 180°
7	Pronate	[19]	Arm on table, pronate 180°
8	AbductShoulder90	[19, 36]	Arm at side, abduct 90°
9	Reach 0 to 1	[36]	Reach from center position to marker “1”
10	Reach 1 to 0	[36]	Return from marker “1” to center
11	Reach 0 to 2	[36]	Reach from center position to marker “2”
12	Reach 2 to 0	[36]	Return from marker “2” to center
13	Reach 0 to 3	[36]	Reach from center position to marker “3”
14	Reach 3 to 0	[36]	Return from marker “3” to center
15	Reach 0 to 4	[36]	Reach from center position to marker “4”
16	Reach 4 to 0	[36]	Return from marker “4” to center
17	Reach 0 to 5	[36]	Reach from center position to marker “5”
18	Reach 5 to 0	[36]	Return from marker “5” to center
19	Shoulder flex 180	[19]	Arm at side, flex shoulder 180°
20	Hand to forehead	[19]	Arm on table, reach up to place hand by forehead
21	Elbow flex 90	[19, 36]	Arm on table, flex elbow 90°
22	Pick up phone	[20]	Arm on table, pick up object in phone answer motion
23	Zip upwards	[20]	Zip upwards from waist to neck
24	Zip downwards	[20]	Zip downwards from neck to waist

**Fig. 1:**
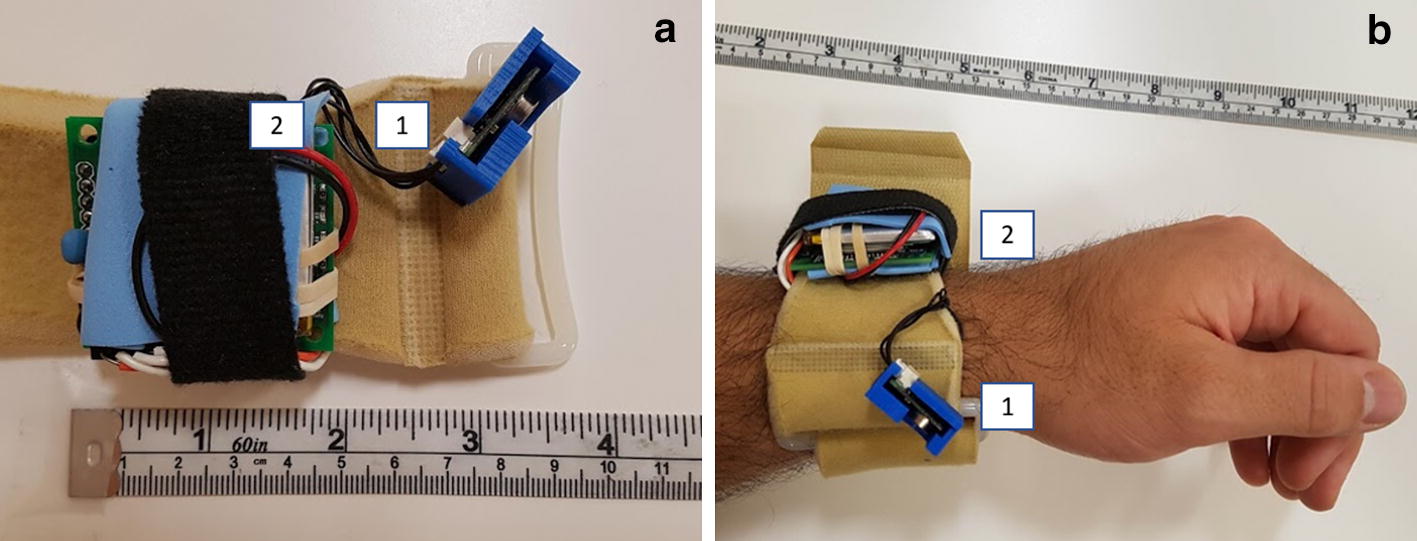
Device images. **a** Device on table, showing thermal sensor (1) and main unit (2). **b** Device positioned on wrist for this study

The device was worn on the left wrist with the thermal sensor located on the radial aspect of the wrist. When the forearm is in neutral pronation, the thermal sensor is oriented at a 45-degree angle such that it is pointing inwards towards the user’s torso, Fig. 1. Sensor orientation was configured to ensure that the user’s torso is in the sensor FOV for most arm motions within a typical user’s range of motion. The device is worn proximal to the radiocarpal joint to remove unwanted influence of the wrist flexion actions.

#### Testing area

**Fig. 2:**
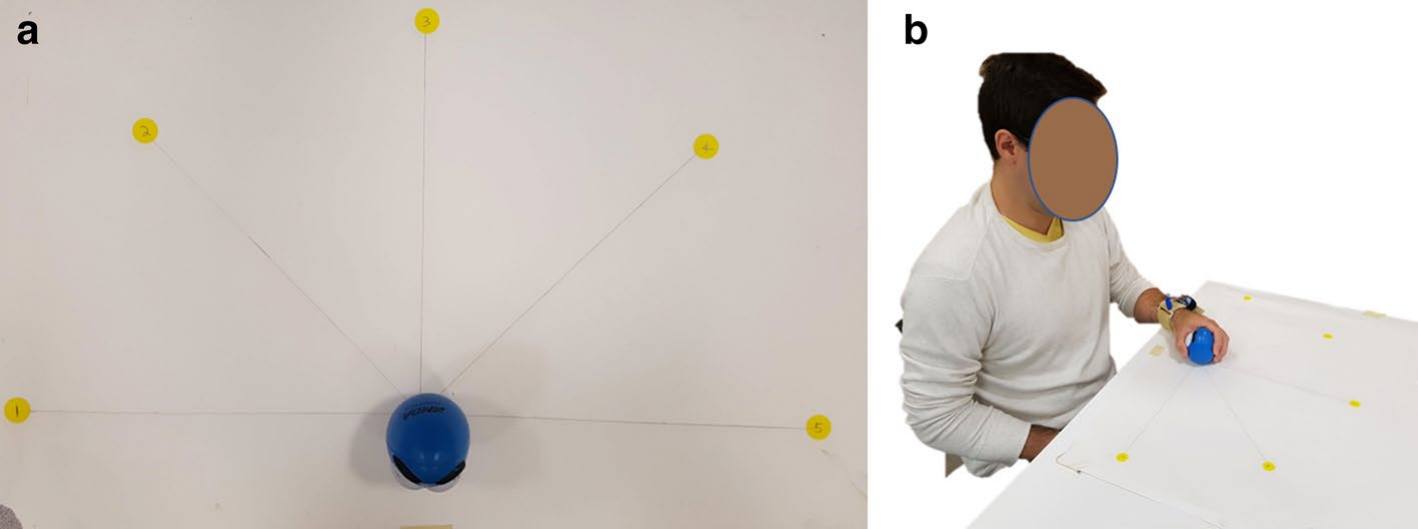
Testing area: **a** 5 positions for reaching task, **b** participant seated for task

Participants completed the protocol while seated in a hard-backed chair in front of a table in a research laboratory (Fig. 2). 5 markers were spaced radially from a central marker on the table as per the GRASP table-top reaching protocol [37], and a soft foam ball was held during these tasks. The outer circles were placed at a 35-cm distance from the central point.

### Data analysis and processing

Data analysis of the system (data cleansing, treatment, and classification) is summarized below. Data were median filtered to smooth high-frequency components before secondary features were calculated for each sensor channel. The 3 point median filter introduces a 17-millisecond delay based on the 12-Hz data recording frequency, but this was not a concern for the moderate speed movements of this study, which were analyzed in an offline model. The resulting data were combined across participants and normalized before training. Each participant completed 24 actions with 10 repetitions, resulting in 240 trials. The secondary features compressed the time series data into a single row per trial, resulting in 240 rows of data per participant. The process of data recording, data treatment, classifier training, and testing is described below.

1. Calculate the spatial average of thermal sensor data.
2. Smoothing: Apply median filter data (window size = 3).
3. Apply secondary features (7 calculations, described in Table 2) to each input channel.

**Table 2:** Summary of secondary features calculated on each sensor channel (primary feature)

#	Secondary feature	Calculation notes
1	Mean	Mean value of each channel for a single motion
2	Standard deviation	Standard deviation of each channel
3	Duration	Number continuous samples above 20th percentile value for the motion
4	Energy	Squared sum of the data sequence
5	Dominant frequency power	The peak power of the Power Spectral Density (PSD), calculated with periodogram function in Matlab
6	Dominant frequency	The peak frequency of PSD
7	Mean power	Average power of PSD
4. Normalize features.
5. Evaluate with different machine learning classifier models. Select an optimal model based on accuracy and processing time.

A description of raw sensor data, calculations, and secondary features is provided below. Raw data included accelerometer, gyroscope, and quaternion from the IMU, as well as thermal data from the Omron D6T sensor. Quaternion is a four-dimensional number composed of a scalar and vector component and is used to represent orientation in 3D space [39]. The quaternion representation of orientation is typically favored over the Euler representation since the Euler representation can suffer from gimbal lock [40] when 90 degree rotations are performed about an axis [41]. Quaternion was calculated by the main microcontroller using the Mahony complementary filter [42] to reduce integration drift in the orientation calculation. Magnetometer data as well as filtered accelerometer and filtered gyroscope sensor data were input to the complementary filter. Raw magnetometer data were not directly used as input to the classification model due to its sensitivity to electronics and metals in environment and its lower 8-Hz update rate. The accelerometer measures in each of its 3 mutually orthogonal sensor axes the vector of the gravitational field and linear acceleration resulting from forces acting on the sensor [38]. The acceleration values recorded from the IMU were normalized relative to the average Earth gravity force of 9.81 m/s^2^. The gyroscope sensor measures angular velocity about each of its 3 mutually orthogonal axes and is converted to degrees per second values for our investigation. Thermal sensor data were spatially averaged in a row-wise, column-wise, and quadrant-wise manner to reduce number of input features to the model and increase processing speed, resulting in 12 primary features from the thermal sensor. Raw accelerometer and gyroscope data were filtered with a 42-Hz digital low-pass filter using the processor integrated in the MPU9250 IMU [33]. 22 primary features were obtained from the 3 axes of accelerometer, 3 axes of gyroscope, 4 quaternions, and 12 values from thermal sensor spatial averages.

**Fig. 3:**
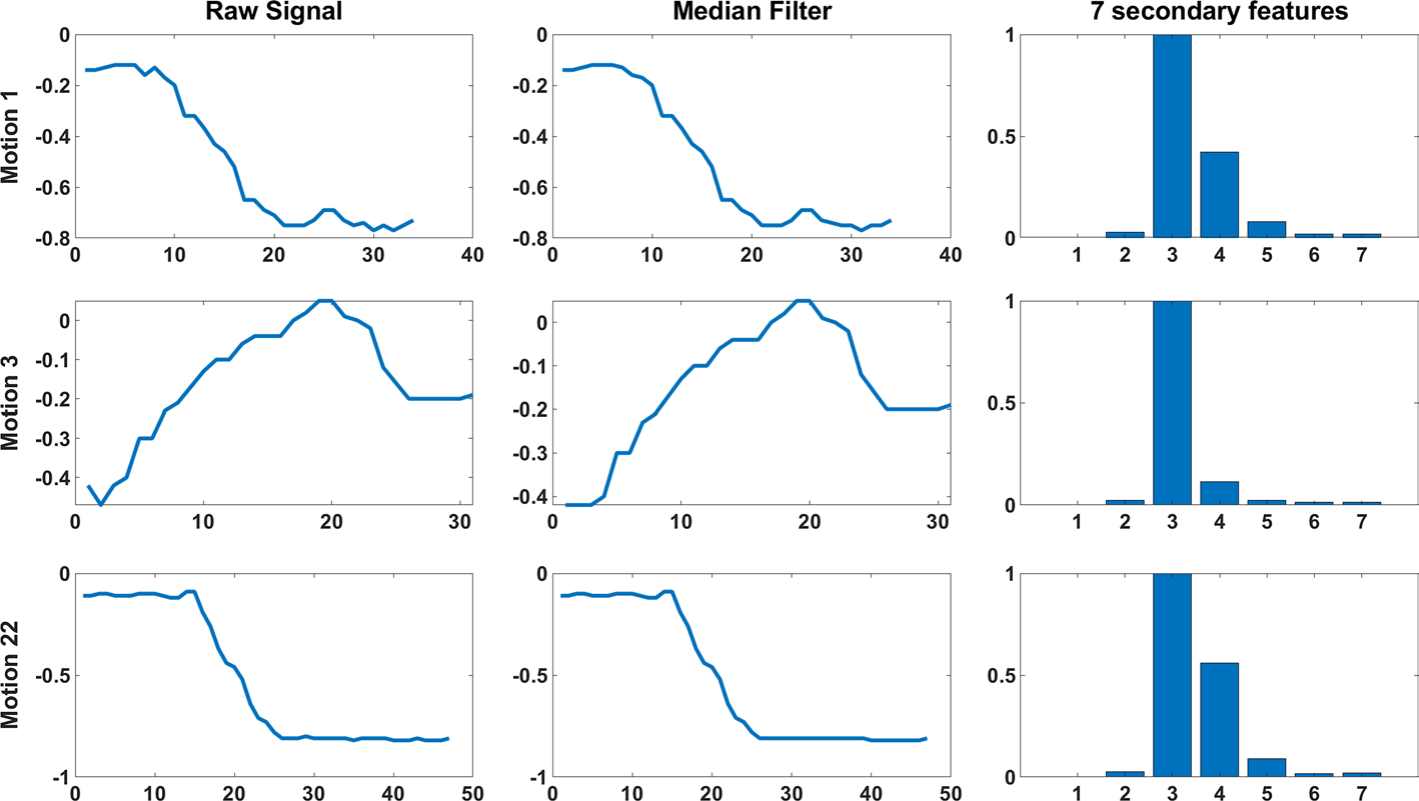
Signal processing steps for 3 different motions. Each row is a different motion. Each column shows a processing step

Seven secondary features were calculated for each of the primary features, Table 2. These secondary features have previously demonstrated strong performance in high-accuracy motion classification in Zhang et al. [18]. These 7 secondary features were applied to each of the 22 primary features, producing 154 secondary features. An example image of feature processing for 3 different motions is shown in Fig. 3; each row is a different motion and each column is a processing step. Note that the median filter will smooth some of the high-frequency components and peaks that are visible in the raw data. Four machine learning models were then applied to the secondary features: Linear Discriminant Analysis (LDA), Support Vector Machine (SVM), K-Nearest Neighbors (KNN), and classification trees. These four machine learning models were selected for analysis because their prevalence in existing motion classification studies allows some aspect of comparison: LDA [43–48], SVM [13, 18, 43, 44, 47, 49]), KNN [13, 18, 47, 48, 50], and Classification trees [8, 13, 51, 52].

### Statistical evaluation

Paired statistical tests were used to assess significance between distributions. Normality of data distributions was assessed with the Shapiro–Wilk test and Q–Q plots where required. Distribution residuals that appeared normal at a *p* = 0.05 significance were tested with a parametric *t* test for single paired test or one-way Analysis of variance (ANOVA) for multiple comparisons. Distributions that did not appear to have normal residuals were analyzed with Wilcoxon signed rank test for single paired test or Kruskal–Wallis for multiple comparisons. Each of these statistical evaluations was completed in Matlab 2018a with a default significance value of *p* = 0.05. Matlab built-in functions ttest, anova1, signrank, and kruskalwallis were used.

## Results

### Model performance

Model comparison was assessed by completing personalized model evaluation in a randomly shuffled, stratified 10-fold cross validation, ensuring that sufficient test data were present in each fold. Machine learning models were evaluated on this dataset and the mean accuracy and processing times were compared, Table 3. Personalized models were evaluated on each participant 10 times by shuffling the data each time, selecting 90% of the data for model training and 10% for model testing. A classification accuracy was reported for each of the 10 iterations and the average classification accuracy for that participant was calculated across the 10 iterations. The overall average classification accuracy was then calculated by taking the average of the 11 participant averages. This was repeated for each of the machine learning models in Table 3. The average time required for the model to classify a set of 24 motions was calculated by averaging the 10 classification times from a participant’s 10 iterations, and then taking the average of the 11 participants’ times.

**Table 3:** Model performance with personalized classification model

Model	Average accuracy, (%)	Average evaluation time to classify 24 motions, (s)
SVM	90.61	4.06
KNN	74.15	0.04
LDA	93.55	0.28
Classification trees	82.08	0.65

Average accuracy is calculated upon the 11 participants’ classification accuracies. Average evaluation time is calculated by taking the average time required to classify a set of 24 motions across the participants

### Performance of personalized models and generalized models

The highest average classification accuracy was 93.55 ± 5.1%, (N = 11), and this was obtained by training personalized LDA classifier models to each participant. A confusion matrix shows the correctly and incorrectly classified actions for each task for the combined results of the personalized model evaluation, (please see Additional file 1). Average precision was 92.1%, average sensitivity (true positive rate, TPR) was 92.0%, and false positive rate (FPR) was 0.4%, (please see Additional file 1).

It was observed that individual classification accuracies for the 10 table-based reaching motions (motions 9–18) were lower than the other 14 motions, with accuracy values of 98.6% and 99.9%, respectively. The performance difference was further observed as a large precision difference (82.8% vs. 98.8%) and a large sensitivity difference, (83.0% vs. 98.4%). As a result, the accuracy of different movement directions was examined. It was found that the performance was significantly correlated to the reaching angle, *F*(4,105) = 48.54, *p* = 4.69e−23. Reaching direction (forward or backward) was not found to significantly correlate to performance, *F*(1,20) = 2.51, *p* = 0.129.

Generalized model classification performance was evaluated as follows. A machine learning model was trained on data from 10 participants, and then the machine learning model was tested on the 11th participant, whose data had not been input into the model. This was completed 11 times, using a different participant for the model testing each time and the remaining 10 participants’ data for training the model. In each iteration, a classification accuracy was calculated for the model’s ability to classify the motions of that participant. Through the 11 iterations, we obtained 11 different classification accuracies and an average classification accuracy of 82.5 ± 10.1% (*N* = 11). A generalized model that can accurately classify a user’s actions without prior training on that user’s data could be beneficial in scenarios where motion classification is needed quickly, or when a user is unable to participate in training the classification model. This could be useful in a rehabilitation scenario where a patient might be unable to complete an entire exercise regimen due to fatigue, yet classification of exercise motions is still desired.

### Sensor comparison

**Fig. 4:**
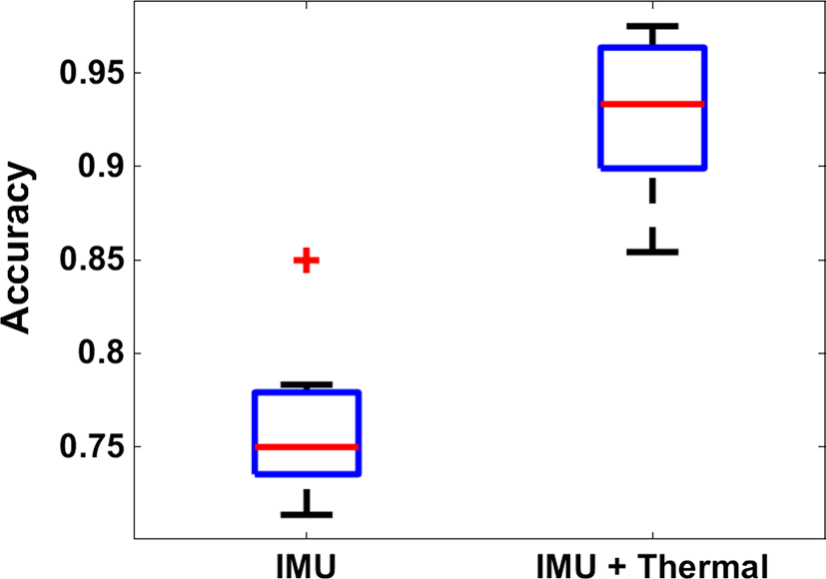
Accuracy comparison for IMU-based secondary features and secondary features from IMU and thermal sensor

The performance contribution of the thermal sensor is explored here. Two datasets were compared: secondary features derived solely from IMU and data features from the IMU and thermal sensor. It was observed that the addition of a thermal sensor contributed to a significant increase in accuracy, from 75 ± 4 to 93.55 ± 4%, (*F*(1,20) = 90.53, *p* = 7.25e−09), Fig. 4.

**Fig. 5:**
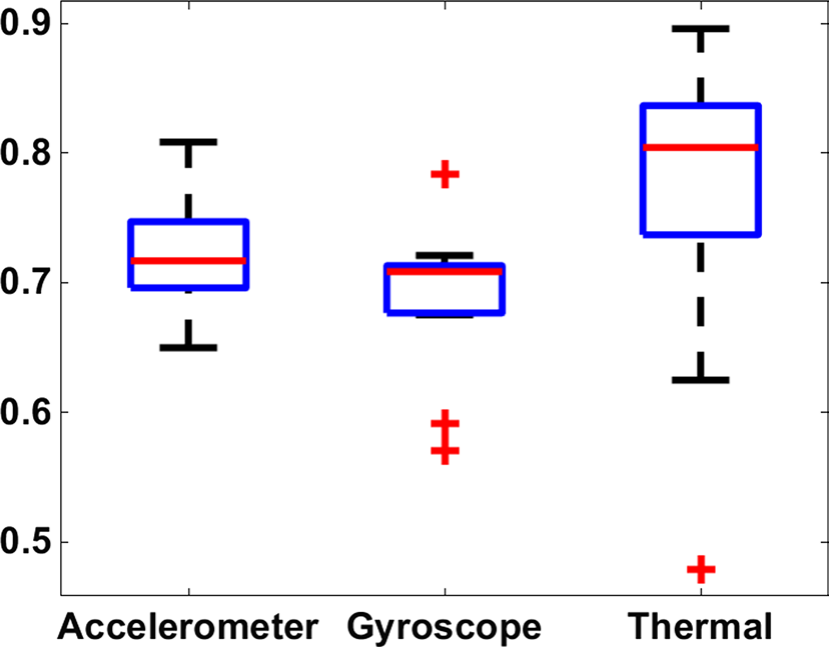
Accuracy from secondary features calculated on each sensor source

Classification accuracy for models trained on secondary features from each sensor (accelerometer, gyroscope, and thermal sensor) were compared to one another. The thermal sensor-based features provided a significantly higher accuracy than gyroscope-based features (*p* = 0.04). Thermal sensor appears to have provided a stronger performance than accelerometer-based features but is not significantly different, (*p* = 0.28), Fig. 5.

## Discussion

### Model performance

The primary criterion for the optimum model is high classification accuracy; however, a short processing time is an additional consideration since a future system might incorporate online classification. LDA was selected as an optimum model due to its highest accuracy and fast processing time relative to the other evaluated models. It was found that KNN had the fastest processing time while delivering poorer classification accuracy and conversely, SVM yielded considerably high classification accuracy but suffered from much slower processing times.

Standard built-in MATLAB models were employed for this analysis, although model parameter tuning was briefly explored. KNN model accuracy was highest for neighborhood size 1, and LDA accuracy was highest with a pseudo-linear discriminant or diagonal linear discriminant instead of a linear discriminant.

Additionally, Principal Component Analysis (PCA) was applied to each model to examine opportunities for a reduced feature space. It was found that PCA could transform the feature space to represent 99.5% of the original variance with a feature reduction from 154 to 60 features with only marginal decrease in accuracy and led to a slightly decreased processing time. Note that classifier performance with the entire feature space should be validated on new populations before feature space reduction is applied, since a different population such as rehabilitation patients might perform exercises differently and with greater variability.

### Performance of personalized models and generalized models

The personalized classification model represents the expected performance for a system that is trained on data from an individual user and then tested on newly recorded data from that individual. This accuracy is expectedly higher than generalized model training, since the data were consistently recorded on the single user with the band worn only once. A rehabilitation patient with sufficient mobility to complete exercises independently in an outpatient rehabilitation session could train a personalized classification model and possibly have their exercise motions classified with a similarly high accuracy. However, it should be noted that the patient’s mechanics of movement may change as they progress through rehabilitation, and the sensor data may change as a result. This system’s classification accuracy compares favorably to accuracies reported in existing work with fewer motions (91–99% accuracy for up to 8 motions, [14, 17, 43]). The confusion matrix shows that a greater number of misclassifications were observed for motions 9–18, which correspond to the table-top reaching exercises (Additional file 1).

The generalized classification model performance was strong considering that the model had not been exposed to any of the participant’s data during training. This indicates that the system has a potential to adapt to new users when the device is worn in a similar manner to the previous users. A system that is used properly could yield up to 82% prediction accuracy on 24 motion classes without any prior training by that user. This performs similarly well to Zinnen et al. [9], which proposed a 2-device system with a device on each wrist; 20 motions were classified in a similar evaluation. They obtained 86% accuracy when a kinematic model constraint was applied, and they obtained 82% without the kinematic model. Comparatively, our 1-device system resulted in a classification accuracy of 93.55% for 24 motions when a personalized classification model was evaluated on each participant, and an accuracy of 82.5% when a generalized classification model was evaluated on participants. The high classification accuracy of the healthy participants’ personalized classification models prompts further investigation into the possibility of training a personalized prediction model for a rehabilitation patient. Future development of this system should also consider use of anatomical constraints and kinematic models for purposes of rejecting unlikely motions and arm poses, and likely increasing classification accuracy.

### Sensor comparison

Results showed that addition of thermal sensor data significantly increased accuracy, Fig. 4. Comparison of accuracy from individual sensors also indicates that the thermal sensor was a strong contributor to accuracy and yielded significantly higher classification accuracy than gyroscope-derived features but was not significantly more accurate than accelerometer-derived features for this study, Fig. 5. While this study applied the same set of feature calculations to each sensor, a future investigation could determine the highest performing features for each sensor and propose specialized features to maximize the contribution of each sensor. Additionally, data rate of the system could be increased if the number of primary features (sensor channels) was reduced or if onboard motion classification was introduced, removing the need for wireless transmission of primary feature data to a processing computer. The increased operating frequency of the system may indeed provide an opportunity to increase classification accuracy; however, Gao et al. [53] and Krause et al. [54] note that the classification accuracies increase marginally with an increased sampling rate and that this can decrease the battery life of the system. Future work will expand on this examination of feature selection and determine the effects of sampling rate on classification accuracy.

### Directional and spatial performance

It was observed that classification accuracy had some correlation to reaching angle. Specifically, movements reaching to the right were most accurate, and movements reaching to the left were least accurate. One possible explanation for this is that reaching to the right with the left hand will cause more of the body to be visible in the thermal sensor FOV, resulting in an improved ability to sense the wrist orientation relative to the body and thus allow more accurate classification of motions. Comparatively, arm movements to the left may move to positions where the thermal sensor cannot detect the participant within its FOV if the participant rotates their shoulder outward during the reaching action. Completing this study with the device worn on the right arm would likely yield the opposite trend.

### Potential clinical implications

Where difficulty to access rehabilitation clinics is a cited barrier to rehabilitation program adherence [22], this technology, once proven successful with a population requiring physical rehabilitation, could improve patient access by allowing patients to participate in rehabilitation at home and track their exercises. Increasing the number of motions that can be classified should increase the opportunity for patients to exercise at home since a wider variety of rehabilitation motions can be practiced.

The increased number of motions that can be classified with this device could possibly lead to an improvement in exercise tracking devices for unsupervised rehabilitation. This is important since inconsistency of patient self-reporting is a recognized challenge [24, 25] and activity tracking devices can be a convenient solution to this self-reporting issue.

Since this technology could potentially facilitate increased usage and acceptance of home-based rehabilitation, it is possible that this technology could assist in reducing costs of healthcare, since home-based rehabilitation has been observed to lower rehabilitation healthcare costs by as much as 15% [23].

Increasing the number of arm rehabilitation motions that can be classified expands the potential benefit that single-device wrist-mounted wearables can bring to home-based rehabilitation monitoring. This study has been able to classify motions from 4 different rehabilitation protocols and we believe this increases the applicability of single-device wrist-mounted wearables for home-based rehabilitation monitoring. In comparison to multi-device systems, this single-device demonstrates a usability improvement since multi-device systems can be more cumbersome to don and would be impractical to wear naturally in a home-based setting.

### Limitations

This was a preliminary study with healthy participants in a controlled environment. It is possible that classification accuracy of this system may decrease in an uncontrolled environment or when used with a different population. Specifically, since stroke impairment can affect smoothness of movement, movement speed, and muscle strength [55, 56], classification of stroke-survivor movements might be more difficult to classify than healthy persons’ motions since these impairments might affect consistency of movement. A rehabilitation patient might also perform the motion differently at different times throughout their rehabilitation, adding further variability to the recorded data.

### Future development

Further development of this system should be applied to more specific scenarios, such as allowing home-based rehabilitation to be more easily monitored by clinicians by facilitating remote access to patient activity data. These sensor arrangements could be developed into customized garments or attached to everyday objects to easily record user motion throughout the home, similar to the work described in Tavares et al. [27], or incorporated into video games to provide motivation and feedback to the participant as described in Bento et al. [57]. Future development should also focus on methods to quantify the quality of motion performed; one such method proposed in Bento et al. [57] defines motion quality by comparing a recorded motion to an expected range of motion.

Integration to secure cloud storage could allow patient exercise data to be delivered remotely to clinical experts. Patient exercise data could be locally recorded through a custom application on an Android or iOS smartphone, which can connect to the device via Bluetooth connection. This information could then be uploaded to a secure online platform via Wi-Fi connection and then accessed by clinical professionals. Future development would be required to develop the smartphone application, integration to a secure file storage service, and user interface for stakeholders to view the patient’s exercise data.

While we were able to achieve a reasonably high classification accuracy on the data that were collected over a period of 3 weeks, future work should examine the performance of the system in long-term operation and examine the effects of sensor drift. Additional filtering or kinematic constraints might be required to maintain robust operation in these situations. The thermal sensor data could provide a useful input to Kalman filter algorithms during long-term operation of the system.

Additionally, future work should further explore and compare the performance of the generalized and personalized classification models. It is possible that a hybrid solution between the personalized and generalized model might provide benefits in a clinical environment. In a hybrid model scenario, a pre-trained, generalized classification model could allow a patient’s motions to be quickly classified with minimal recording of training data, and the model could become personalized over time by incorporating data from the patient.

## Conclusion

This paper evaluated the possibility of increasing the classification accuracy of an IMU-based single-device wearable by adding a thermal sensor. Our results indicate that the thermal sensor significantly increased the classification accuracy of a single-device system that was evaluated on 24 arm motions in a study with 11 healthy people. This single-device system for arm motion classification exceeded the number of arm motions classified by previously presented systems found in the literature. This system demonstrated 93.55% accuracy with a personalized classification model and 82.5% accuracy with a generalized model. The high accuracy with which this system classified arm motions prompts the need for future evaluation for specific applications, such as classifying exercises of stroke-affected individuals in controlled environments and home environments.

## Additional file

**Additional file 1.** Confusion matrix and classification statistics.

## Data Availability

The datasets used and analyzed during the current study are available from the corresponding author on reasonable request.

## Abbreviations

FOV: field of view
IMU: inertial measurement unit
PSD: power spectrum density
