# Supplementary material for: Would a thermal sensor improve arm motion classification accuracy of a single wrist-mounted inertial device?
Source: Biomed Eng Online. 2019 May 7;18:53. doi: 10.1186/s12938-019-0677-7 (PMC6505300; doi:10.1186/s12938-019-0677-7)
Supplement: Supplementary file 1 — Additional file 1. Confusion matrix and classification statistics. [file 12938_2019_677_MOESM1_ESM.docx]

**Additional File 1 – Confusion Matrix and classification statistics**

This file displays the confusion matrix for 24 types motions as performed by 11 participants in 11 trials. The actual motion performed is viewed in the x axis (Target) while the motion predicted by the classifier is displayed in the corresponding row. The colour map also indicates the relative score of each cell, on a scale from 0 to 110. Since 11 participants performed each motion 10 times each, a perfect score for that motion would be observed as a value of 110 in the diagonal of this matrix.


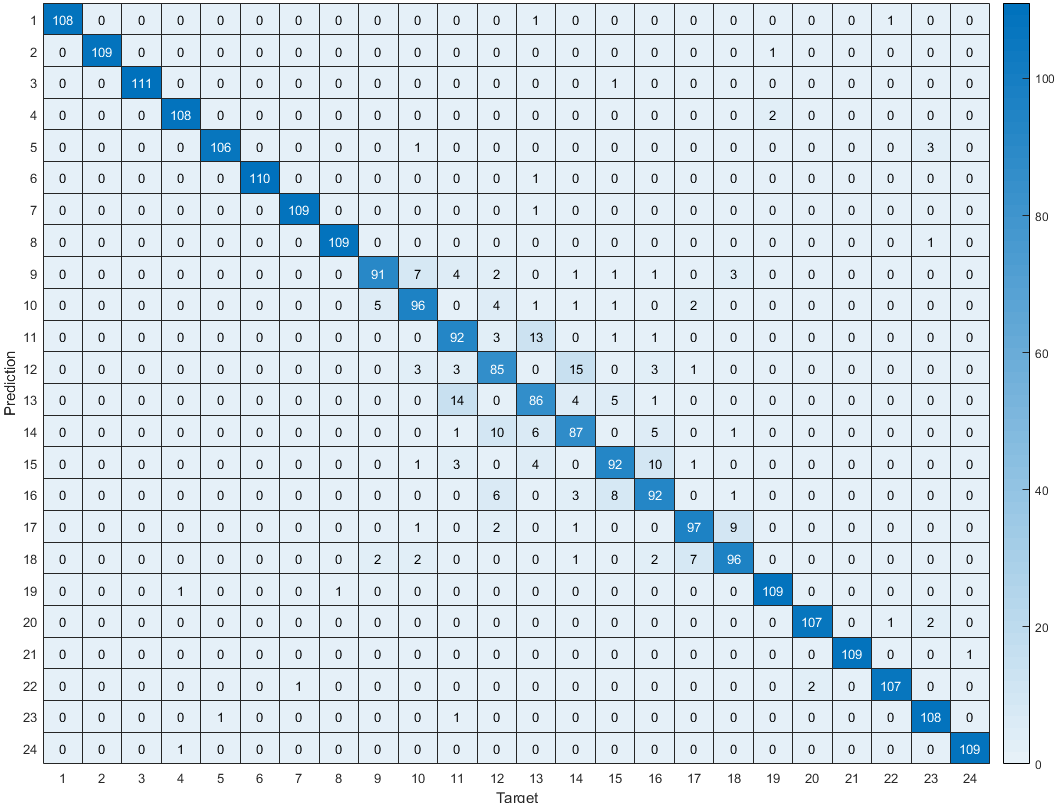


The table below shows additional statistics associated with the classifier performance for each of the 24 motions. Please note the following acronyms: TP = True positive, FP = False positive, TN = True negative, FN = False negative. True positive (TP) represents the number of true predictions when the Target is true, and matches the values in the diagonal of the confusion matrix. Accuracy is calculated as (true positive + true negative)/total. Precision is calculated as (TP)/(TP + FP). Sensitivity or True Positive Rate (TPR) is calculated as (TP)/(TP + FN). False Positive Rate (FPR) is calculated as (FP)/(FP + TN).

Please also note that motions 9-18 correspond to table-based reaching motions. Since performance was lower for these motions, average performance statistics were computed separately for this group.

| Motion # | True Positive | Accuracy | Precision | Sensitivity, or True Positive Rate | False Positive Rate |
| --- | --- | --- | --- | --- | --- |
| 1 | 108 | 0.9992 | 1.0000 | 0.9818 | 0 |
| 2 | 109 | 0.9996 | 1.0000 | 0.9909 | 0 |
| 3 | 111 | 0.9996 | 1.0000 | 0.9911 | 0 |
| 4 | 108 | 0.9985 | 0.9818 | 0.9818 | 0.0008 |
| 5 | 106 | 0.9981 | 0.9907 | 0.9636 | 0.0004 |
| 6 | 110 | 0.9996 | 1.0000 | 0.9910 | 0 |
| 7 | 109 | 0.9992 | 0.9909 | 0.9909 | 0.0004 |
| 8 | 109 | 0.9992 | 0.9909 | 0.9909 | 0.0004 |
| 9 | 91 | 0.9902 | 0.9286 | 0.8273 | 0.0028 |
| 10 | 96 | 0.9890 | 0.8649 | 0.8727 | 0.0059 |
| 11 | 92 | 0.9834 | 0.7797 | 0.8364 | 0.0103 |
| 12 | 85 | 0.9803 | 0.7589 | 0.7727 | 0.0107 |
| 13 | 86 | 0.9807 | 0.7611 | 0.7818 | 0.0107 |
| 14 | 87 | 0.9815 | 0.7699 | 0.7909 | 0.0103 |
| 15 | 92 | 0.9864 | 0.8440 | 0.8288 | 0.0067 |
| 16 | 92 | 0.9845 | 0.8000 | 0.8364 | 0.0091 |
| 17 | 97 | 0.9909 | 0.8981 | 0.8818 | 0.0043 |
| 18 | 96 | 0.9894 | 0.8727 | 0.8727 | 0.0055 |
| 19 | 109 | 0.9981 | 0.9732 | 0.9820 | 0.0012 |
| 20 | 107 | 0.9981 | 0.9817 | 0.9727 | 0.0008 |
| 21 | 109 | 0.9996 | 1.0000 | 0.9909 | 0 |
| 22 | 107 | 0.9981 | 0.9817 | 0.9727 | 0.0008 |
| 23 | 108 | 0.9970 | 0.9474 | 0.9818 | 0.0024 |
| 24 | 109 | 0.9992 | 0.9909 | 0.9909 | 0.0004 |
| **Mean, overall** | 101 | 0.9933 | 0.9211 | 0.9198 | 0.0035 |
| Mean, motions 9-18 | 91 | 0.9856 | 0.8278 | 0.8302 | 0.0076 |
| Mean, motions 1-8 and 19-24 | 109 | 0.9988 | 0.9878 | 0.9838 | 0.0005 |
